# Gene mutations in sporadic lymphangioleiomyomatosis and genotype–phenotype correlation analysis

**DOI:** 10.1186/s12890-022-02154-0

**Published:** 2022-09-18

**Authors:** Jiannan Huang, Wenshuai Xu, Peng Liu, Yaping Liu, Cheng Shen, Song Liu, Yani Wang, Jun Wang, Tengyue Zhang, Yudi He, Chongsheng Cheng, Luning Yang, Weihong Zhang, Xinlun Tian, Kai-Feng Xu

**Affiliations:** 1 Department of Pulmonary and Critical Care Medicine, Peking Union Medical College Hospital, Chinese Academy of Medical Sciences and Peking Union Medical College, Beijing, 100730 China; 2 State Key Laboratory of Complex Severe and Rare Diseases, Peking Union Medical College Hospital, Chinese Academy of Medical Sciences and Peking Union Medical College, Beijing, 100730 China; 3 Medical Science Center, Peking Union Medical College Hospital, Chinese Academy of Medical Sciences and Peking Union Medical College, Beijing, 100730 China; 4 Department of Medical Genetics, School of Basic Medicine, Chinese Academy of Medical Sciences, Peking Union Medical College, Beijing, 100730 China; 5 Department of Radiology, National Center of Gerontology, Beijing Hospital, Beijing, 100005 China; 6 Department of Radiology, Peking Union Medical College Hospital, Chinese Academy of Medical Sciences and Peking Union Medical College, Beijing, 100730 China

**Keywords:** Lymphangioleiomyomatosis, *TSC2*, *VEZF1*, Phenotype, Severity

## Abstract

**Background:**

Sporadic lymphangioleiomyomatosis (S-LAM) is a rare neoplasm with heterogeneous clinical features that is conventionally considered to be related to *TSC2*. This study serves to elucidate the mutation landscape and potential correlation between S-LAM genomic profiles and clinical phenotypes.

**Methods:**

Genomic profiles of 22 S-LAM patients were obtained by sequencing genomic DNA and cell-free DNA from various specimens using an NGS (next-generation sequencing)-based tumor-driver gene panel. Detected mutations were summarized. Symptoms, serum vascular endothelial growth factor D (VEGF-D) values, pulmonary function, and six-minute walk distance (6MWD) were compared among groups with different *TSC2* status and genotypes to analyze genotype–phenotype correlations.

**Results:**

67 Variants in 43 genes were detected, with a *TSC2* mutation detection rate of 68.2%. The *TSC2* detection rate was similar in specimens obtained either through transbronchial lung biopsy (TBLB) or surgical lung biopsy (70.0% vs. 69.2%, *p* > 0.05). A novel mutation in *VEZF1* (c.A659G) was detected in four participants and may represent a mild disease state. *TSC2* mutation was significantly related to a shorter 6MWD (*p* < 0.05), and a higher percentage of VEGF-D over 800 pg/mL (*p* < 0.05); stop-gain mutation was significantly related to a higher prevalence of pneumothorax.

**Conclusions:**

Tumor-driver mutations in genes other than *TSC2* may have a role in S-LAM, and TBLB specimens are practical alternatives for genomic analysis. *TSC2* mutation detectability and types are related to the disease severity and phenotypes of S-LAM.

**Supplementary Information:**

The online version contains supplementary material available at 10.1186/s12890-022-02154-0.

## Introduction

Lymphangioleiomyomatosis (LAM) is a rare, progressive, multisystem neoplasm that predominantly affects women [1, 2]. LAM occurs in approximately 3.4–7.8 per million women [3] and is characterized by proliferation and invasion of smooth muscle-like cells (LAM cells), leading to tissue destruction [4] and symptoms that involve various systems. Typical presentations include dyspnea, recurrent pneumothorax, chylous effusions, retroperitoneal/pelvic lymphangioleiomyomas, and renal angiomyolipomas. Accelerated decline in pulmonary function [2] and bilateral cystic lung lesions on imaging [5, 6] are also common findings. LAM can occur either sporadically or concomitantly in patients with tuberous sclerosis complex (TSC), an autosomal dominant disease caused by *TSC1*/*TSC2* gene inactivation. More than 80% of LAM cases are the sporadic form (S-LAM) [7].

S-LAM is conventionally considered to be a disease solely associated with the *TSC2* gene. Multiple modalities of *TSC2* alterations, including loss of heterozygosity (LOH), somatic mutations, rearrangements, and deletions [8–12], have been detected in S-LAM patients [8, 9, 13, 14]. Nevertheless, *TSC2* mutations are not found in every S-LAM patient. LOH analysis has revealed functional *TSC2* alterations in 90%, 80%, 69%, and 50% of peripheral blood, bronchoalveolar lavage fluid (BALF), urine, and chyle samples, respectively [13]. In S-LAM, the detection rate of *TSC2* mutation based on NGS-based techniques is 60–80% [10–12, 15].

Mutations in genes other than *TSC2* are actively being investigated. However, the other common culprit in TSC, *TSC1* mutation, is conventionally considered to have no role in S-LAM. In 2002, a study analyzing *TSC1* and *TSC2* in LAM reported a *TSC1* mutation in an S-LAM patient [16], though the diagnosis was later confirmed to be a forme fruste of TSC rather than S-LAM [17]. More recent studies have also described several cases of *TSC1* alterations in S-LAM [12, 14, 15]. Research using various methods, such as NGS, immunohistochemistry followed by in vivo transgenic mouse experiments, genome-wide association studies, and single-cell and single-nucleus sequencing, have revealed many potential causal genes in LAM, including *PPP2R2B* [12], *HMGA2* [18], *PMEL* [14], and *NR2F2* [19], among others. Nevertheless, whether any of the mutated genes discovered have roles as drivers in S-LAM has not been well supported by exhaustive mechanistic studies.

S-LAM exhibits high heterogeneity [4], and one of the challenges for understanding LAM is precise discrimination, which would allow intricate and individualized treatment approaches. Although researchers have been able to relate a subset of *CCL2* gene polymorphisms to a declining forced expiratory volume in 1 s (FEV_1_) rate [20], and LOH were more likely to be detected in the urine of patients with AMLs than those without AMLs[13], data on genotype–phenotype correlation in S-LAM is scarce.

In this study, we applied NGS-based techniques to obtain the tumor-driver gene mutational profiles of S-LAM patients. We hypothesize that *TSC2* mutations would be detected in most but not all S-LAM patients but that *TSC1* mutations would be absent. We also aimed to explore the potential relationship between *TSC2* mutational profiles and phenotypes.

## Materials and methods

### Patients and samples

Eligible S-LAM patients were enrolled consecutively from 29th October 2015 to 3rd March 2017 from the Peking Union Medical College Hospital (PUMCH) clinic. The enrollment criteria included the following: (1) received a definitive clinical diagnosis of S-LAM; (2) had either planned to undergo a TBLB procedure or had undergone surgical procedures with obtainable and pathologically confirmed LAM-related formalin-fixed paraffin-embedded (FFPE) tissue samples; (3) had not been treated with rapamycin; (4) participation in the study was agreed upon, and informed consent was signed.

A definitive clinical diagnosis of S-LAM was established when a patient with a compatible clinical history and multiple characteristic bilateral cysts on chest high-resolution computed tomography (HRCT) displayed at least one of the following: (1) renal angiomyolipoma (AML); (2) serum VEGF-D ≥ 800 pg/ml; (3) chylous effusions; (4) lymphangiomyomas; (5) histopathological diagnosis; (6) no evidence of tuberous sclerosis complex (TSC) according to detailed history inquiries and physical examinations.

TBLB-acquired or surgically acquired specimens were collected. The TBLB procedures were completed at PUMCH, and fresh specimens were collected. The surgical specimens were obtained by various biopsy procedures, including video-assisted thoracoscopy, nephrectomy, and retroperitoneal/pelvic mass resection at different health-care facilities. All retrieved FFPE specimens were obtained within two years after biopsy. Peripheral venous blood samples were collected from each patient.

### Targeted tumor-driver gene sequencing

Genomic DNA was extracted from tissue specimens and peripheral blood mononuclear cells (PBMCs) using standard methods. A commercially available gene panel—Mygenostics Tumor Driver (MyGenostics, Beijing, China)—was applied to perform targeted DNA sequencing. Briefly, targeted genes were enriched using a biotinylated capture probe (MyGenostics, Baltimore, MD, USA) [21] and sequenced using the Illumina NextSeq500 platform (Illumina, San Diego, CA, USA). The panel contained 301 tumor-driver genes with an average effective sequencing depth above 1000X. A complete list of the tested genes is provided in Additional file 1. Circulating cfDNA was extracted using Magnetic Serum/Plasma DNA Maxi Kit (Tiangen Biotech, Beijing, China). Barcode-assisted target enrichment was performed, followed by polymerase chain reaction (PCR). The same 301 genes as the panel used for tissue were sequenced with the Illumina NextSeq500 platform.

### Vascular endothelial growth factor D (VEGF-D), 6-minute walk distance, and pulmonary function test (PFT)

The serum level of VEGF-D was detected with an enzyme-linked immunosorbent assay (ELISA, R&D Systems, Minneapolis, MN, USA), and procedures were performed according to the manufacturer’s instructions at the Laboratory of Respiratory Diseases of PUMCH. Evaluation of the six-minute walk test (6MWT) was performed abiding according to the ATS procedures [22]. To avoid pneumothorax-related complications or inaccurate results, all patients who underwent a pulmonary function test were either free of pneumothorax or had a time lag between PFT and resolved pneumothorax of at least three months.

### Statistical analysis

Continuous variables are described as the mean ± standard deviation. Means of different groups were compared using Student’s t-test (default) or Welch’s t-test. A homogeneity test of variance is performed for each analysis set to measure the comparability between different groups, ensuring that the chosen statistical tests for different groups are appropriate for the distribution of data. More specifically, Welch's t-test is used when the homogeneity test of variance showed a significant difference, while Student's t-test is used to compare groups with comparable variance. A multivariable linear regression and a hierarchical multivariable linear regression were performed to analyze the influence of various factors on clinical parameters. The ratio of discontinuous variables was compared using the Chi-squared test (default) or Fisher’s exact test (when any number in a crosstab was five or less). All calculations were performed by using IBM SPSS Statistics 25 [23].

## Results

### Characteristics of the patients and samples

A total of 22 subjects were enrolled in this study. The demographics and clinical features of the patients are listed in Table 1, and detailed clinical information for each patient is provided in Additional file 2. Twenty-three tissue samples [see Additional file 3] were obtained, 10 (43.5%) of which were lung tissue acquired by TBLB. Another 6 (26.1%) samples were FFPE lung tissue obtained by video-assisted thoracoscopy (VATS), 4 (17.4%) were from FFPE extraperitoneal masses, and 3 (13.0%) were from FFPE renal AML. Of note, patient LAM3 underwent both VATS for lung biopsy and nephrectomy surgery to remove left-side renal AML, and the tissue resected in both surgeries was sequenced in this study.

**Table 1:** Demographics and clinical features of the enrolled LAM patients

Characteristics	Ratio (%) or median (range) or mean ± SD
*Sex*	
Female, n (%)	22 (100.0%)
*Ethnic*	
Asian, n (%)	22 (100.0%)
^a^Age, median (range) (year)	38.5 (25–57)
Postmenopause, n (%)	2 (9.1%)
Smoking history, n (%)	0 (0.0%)
Pneumothorax, n (%)	11(50.0%)
Chylous effusions, n (%)	3 (13.6%)
Renal angiomyolipoma, n (%)	4 (18.2%)
^b^Lymphangioleiomyomas, n (%)	16 (72.7%)
^c^Rapamycin therapy, n (%)	0 (0.0%)
Serum VEGF-D, mean ± SD (pg/ml)	1719.0 ± 1311.4
FEV_1_% pred, mean ± SD (%)	76.3 ± 19.0
DL_CO_ % pred, mean ± SD (%)	59.8 ± 22.9
6MWD (meters)	493.1 ± 86.1
CT grading	
Grade I, n (%)	5 (22.7%)
Grade II, n (%)	7 (31.8%)
Grade III, n (%)	10 (45.5%)

*SD* standard deviation, *VEGF-D* vascular endothelial growth factor-D, *FEV*_*1*_*%pred* forced expiratory volume in first second percent predicted, *DL*_*CO*_* %pred* diffusing capacity of carbon monoxide percent predicted, *6MWD* six-minute walk distance, *6MWD* six-minute walk distance

^a^Age: the age when the patient was diagnosed

^b^All the CT results were examined by a radiology specialist to determine the presence of retroperitoneal or pelvic masses and to confirm whether the characteristics of the masses were compatible with lymphangioleiomyoma

^c^All patients were rapamycin treatment-naïve at the time their blood samples were collected

### Mutation profile

**Fig. 1:**
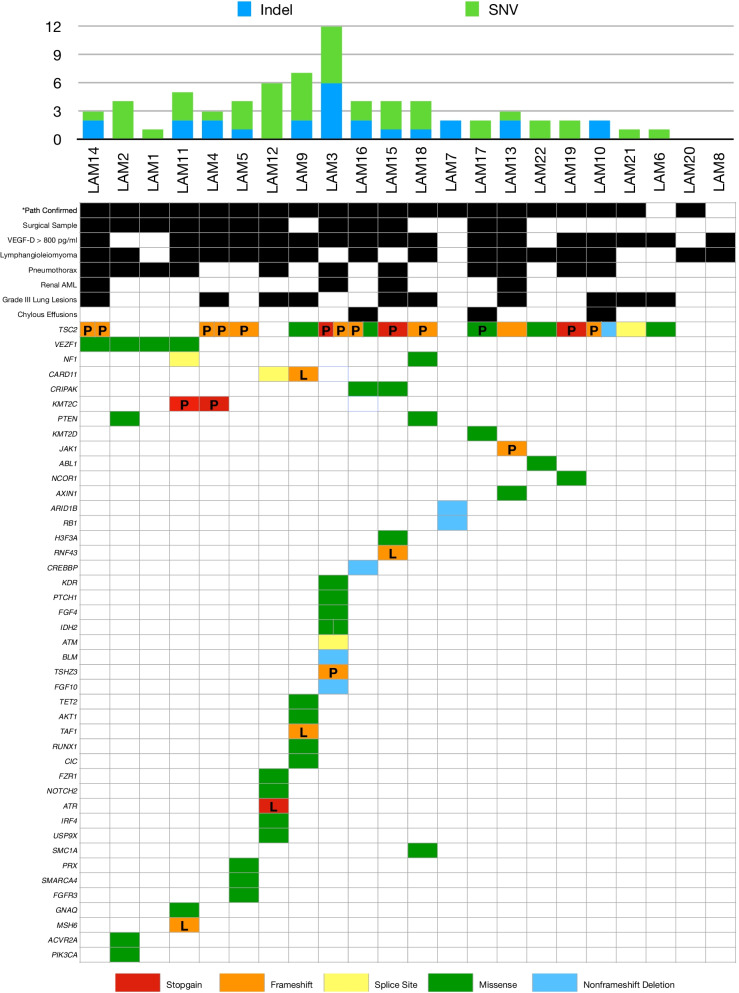
Mutated genes and clinical features, including specimen information, symptoms, VEGF-D values, and the grade of lung lesions. The patient number (*x*-axis) and their corresponding features (*y*-axis) are shown. Clinical features are in black. Mutated genes with different mutation function types are shown in various colors, including red, orange, yellow, green and blue. *Path confirmed = Histopathologically confirmed LAM specimen; VEGF-D = vascular endothelial growth factor-D; AML = angiomyolipoma. The pathogenicity of each mutation is represented by P = pathogenic; L = likely pathogenic; and a blank cell = uncertain function. Mutation function annotation was provided by MyGenostics Co. following the American College of Medical Genetics and Genomics (ACMG) guidelines

**Fig. 2:**
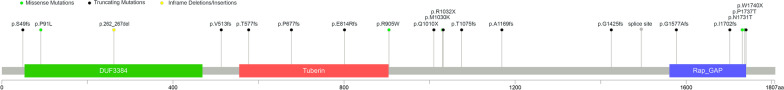
Schematic diagram of detected *TSC2* gene mutations. A total of 20 mutations in TSC2 are depicted. All mutations were found only once. The amino acid changes are labeled. The stop-gain (also known as nonsense) and frameshift mutation function types lead to truncated protein products. DUF3384, tuberin, and Rap_GAP are three conserved domains of TSC2

Altogether, 67 somatic mutations in 43 genes were found in this study. The mutated genes are presented in Fig. 1; a complete list of the mutation details is provided in Additional file 4. Twenty different mutations in *TSC2* were detected; the specifics were mapped using the MutationMapper package [24], as shown in Fig. 2.

The overall detection rate of *TSC2* mutations was 68.2% (15/22), and the *TSC2* mutation detection rate was 71.4% (15/21) among those with a pathohistologically confirmed LAM. Counterintuitively, a detection rate comparable to that of surgical specimens was found for TBLB samples. Specifically, *TSC2* mutation was detected in 7 of the 10 (70%) TBLB samples but in 69.2% (9/13) of surgically acquired samples.

According to cfDNA sequencing, 8 mutations in 8 genes (*SMARCA4, PRX, FGFR3* in Patient LAM5, *TSC2, RUNX1, CIC* in Patient LAM9, *FGF10* in Patient LAM3, *MSH6* in Patient LAM11) were detected, with a *TSC2* mutation detection rate of 4.5% (1/22). The mutation profile based on cfDNA was not consistent with that based on tissue samples, and only two mutations (*FGF10* in LAM3 and *MSH6* in LAM11) were repeatedly found in both.

Other than *TSC2*, most genes with mutation were detected only once. *TSC1* mutation was not found. Mutations in *VEZF1, NF1, CARD11, CRIPAK, KMT2C,* and *PTEN* were observed multiple times in at least two subjects. Notably, a specific mutation in *VEZF1* (Transcript NM_007146, nucleotide change c.659A > G, protein change p.H220R) was found in four patients (LAM1, LAM2, LAM11, and LAM14). Table 2 describes the characteristics of the four patients with *VEZF1* mutation. Additionally, an identical *KMT2C* mutation (Transcript NM_170606, nucleotide change c.3168G > A, protein change p.W1056X, stop-gain) was found in both patients LAM4 and LAM11.

**Table 2:** Characteristics of patients with an identical *VEZF1* mutation c.A659G

	LAM1	LAM2	LAM11	LAM14
^a^Age	25	29	30	45
*TSC2* mutation				x
Chyle				
Renal AML				x
Lymphangioleiomyomas		x	x	x
Pneumothorax	x	x	x	x
CT grade	I	II	II	III
FEV_1_%	75.8	73.6	82.6	54.8
DL_CO_ %	71.8	77	80.6	25.1
6MWD (meters)	531	589	540	333
VEGF-D (pg/ml)	710.3	322.9	2237.4	3192.4

*AML* angiomyolipoma, *FEV*_*1*_*%pred* forced expiratory volume in first second percent predicted, *DL*_*CO*_* %pred* diffusing capacity of carbon monoxide percent predicted, *6MWD* six-minute walk distance, *6MWD* six-minute walk distance, *VEGF-D* vascular endothelial growth factor-D

^a^Age: the age when the patient was diagnosed

### Analyzing the relationship between genotype and phenotype

**Fig. 3:**
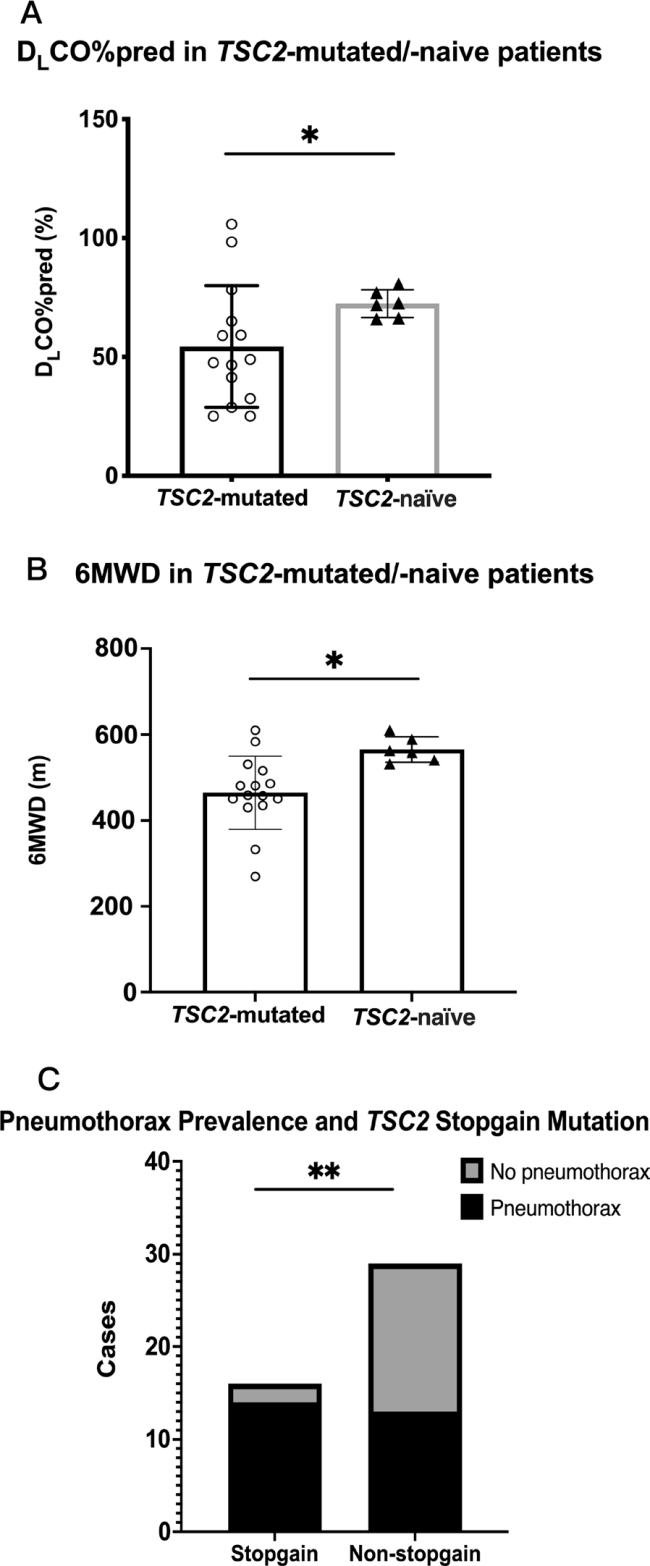
Correlation of *TSC2* mutation status/function type and DL_CO_ %pred, 6MWD, and pneumothoraces. **A** The average DL_CO_ %pred in the TSC2-mutated group was 54.39% versus 72.36% in the TSC2-naïve group (Welch’s t-test, *p* = 0.0243). **B** The average 6MWD in the TSC2-mutated group was 465.1 m versus 564.83 m in the TSC2-wildtype group (Student’s t-test, *p* = 0.0115). **C** The prevalence of pneumothorax in patients with a stop-gain TSC2 mutation was 87.5% (14/16) versus 44.8% (13/29) in patients with other mutation types (Chi-squared test, *p* = 0.0098). DL_CO_ %pred = diffusing capacity of carbon monoxide percent predicted; 6MWD = six-minute walk distance. * indicates* p *< 0.05, ** indicates *p *< 0.01

We compared various disease severity-related parameters, including FEV_1_ percent predicted (FEV_1_%pred), DL_CO_ percent predicted (DL_CO_ %pred), 6MWD, and VEGF-D values, in patients with or without a detected *TSC2* mutation. Considering that age and time with symptom may influence pulmonary function test results and 6MWD, we also conducted multivariable analysis of the effect of age and time with symtom on clinical parameters. Note that time with symptoms was defined as the time between first symptom onset and the first comprehensive evaluation in our center, when PFT, VEGF-D study, and 6MWD study were performed. In the preliminary two sample t-test analysis, we found that DL_CO_ %pred and 6MWD in the *TSC2*-mutated group were significantly lower than those in the *TSC2*-wildtype group (Fig. 3). A following multivariable analysis verified the correlation of a lower 6MWD and *TSC2* mutation (*p* < 0.05), while DL_CO_ %pred was lower in *TSC2*-mutated group with no statistical significance (*p* = 0.06) (Table 3). We then performed a hierarchical multivariable linear regression to verify how *TSC2* mutation status influnce 6MWD on top of other independent variables including PFT, VEGF-D, age, and time with symptom. The R^2^ change was 0.162 after adding *TSC2* into the predictive model with a significant F change of 0.031 (*p* < 0.05), indicating that *TSC2* mutation positivity has a statistically significant negative correlation with 6MWD.

**Table 3:** Multivariable linear regression analysis between clinical parameters and *TSC2* mutation, adjusted by age when diagnosed and time with symptoms

	SE	Beta	*p*-value	Multiple R^2^
*6MWD*				
Constant	88.545	–	0.000	0.639
*TSC2*	30.358	− 0.484	*0.031	
^a^Age	1.697	− 0.596	*0.013	
^b^Onset	0.293	0.375	0.114	
^c^FEV_1_	1.042	0.426	0.151	
VEGF-D	0.010	− 0.012	0.946	
^d^DL_CO_	0.871	− 0.222	0.444	
*DL*_*CO*_				
Constant	59.873	–	0.385	0.664
*TSC2*	9.859	− 0.417	0.060	
^a^Age	0.653	− 0.236	0.355	
^b^Onset	0.091	0.374	0.101	
FEV_1_	0.232	0.793	*0.001	
6MWD	0.084	− 0.206	0.444	
VEGF-D	0.003	− 0.016	0.928	
*FEV*_*1*_				
Constant	47.799	–	0.514	0.698
*TSC2*	7.724	0.402	0.055	
^a^Age	0.509	0.256	0.289	
^b^Onset	0.066	− 0.454	*0.029	
DL_CO_	0.144	0.713	*0.001	
6MWD	0.063	0.357	0.151	
VEGF-D	0.002	− 0.112	0.504	
*VEGF-D*				
Constant	5557.114	–	0.398	0.159
*TSC2*	1048.88	0.107	0.776	
^a^Age	62.085	− 0.190	0.643	
^b^Onset	9.274	− 0.162	0.668	
FEV_1_	32.045	− 0.312	0.504	
DL_CO_	25.693	− 0.040	0.928	
6MWD	7.990	− 0.029	0.946	

*6MWD* six-minute walk distance, *VEGF-D* vascular endothelial growth factor-D, *SE* standard error

^a^Age = age when the patient was diagnosed

^b^Onset = time with symptom

^c^FEV_1_ = forced expiratory volume in first second percent predicted

^d^DL_CO_ = diffusing capacity of carbon monoxide percent predicted

To explore whether clinical symptoms are related to the presence and types of *TSC2* mutation, we compared the prevalence of certain symptoms between different *TSC2* statuses; to achieve higher statistical power, we reviewed and extracted available data from the literature regarding LAM genomic studies. Only one study (Liu et al., 2019) [21] provided both *TSC2* mutation information and corresponding clinical data, and the clinical characteristics of the S-LAM patients in Liu’s study were compared with those in the present study [see Additional file 5_sheet1]. Overall, a statistically significant difference was observed only in the prevalence of retroperitoneal/pelvic lymphangioleiomyomas, which is a less studied LAM sign and is often underdiagnosed. In our study, a Radiology specialist provided an independent second opinion on abdominal and pelvic CT scans to determine the presence of lymphangioleiomyomas in each patient. Nonetheless, all the other patient characteristics were similar in the two studies, and we consider the data in Liu’s study to be comparable to ours. The *TSC2* mutation profile and symptomatic information of 45 clinically and genetically confirmed S-LAM patients were extracted from Liu’s work and combined with our cohort [22 subjects].

In general, stop-gain mutation in *TSC2* was related to a higher pneumothorax prevalence (Fig. 3). Additionally, subjects with detectable *TSC2* mutations were more likely to have a VEGF-D value over 800 pg/mL than were those without a detectable *TSC2* mutation (77.8% vs. 50%, Chi-squared test, p < 0.05). The remaining statistical analysis results are provided in Additional file 5.

## Discussion

### Sequencing strategy and *TSC2* mutation detection rate and profile

Strategies in LAM genomic studies have been evolving over the past two decades. In 1998, Smolarek et al. reported that seven of thirteen (54%) renal AML patients with S-LAM had *TSC2* LOH [8]. Carsillo et al. applied single-strand conformation polymorphism (SSCP) analysis to show that five of seven (71%) renal AML patients carried somatic *TSC2* mutations [9]. To ensure that most of the DNA analyzed originated from cells of the lesion instead of surrounding normal cells, the laser capture microdissection method has been utilized in most LAM genetic studies [9–12, 16]. Furthermore, research over the past ten years has used surgically acquired lung tissue and incorporated the NGS technique for sequencing, with a *TSC2* mutation detection rate of 60–80% [10–12]. More contemporary studies have employed other methods of sequencing and used circulating cfDNA as the material. In 2019, Zhang et al. sequenced cfDNA in 23 LAM patients but found no *TSC2* mutation [14]. Additionally, Liu et al. employed multiple sequencing methods, including target capture sequencing, Sanger sequencing, chromosomal microarray analysis (CMA), and multiplex ligation-dependent probe amplification (MLPA), to reveal a *TSC2* mutation detection rate of 70.6% (36/51) in S-LAM subjects [15]. In the recent single-cell/nucleus sequencing study by Guo et al., all four LAM patients examined were confirmed to carry *TSC2* mutations (100%, 4/4) [25].

In this study, we explored both tissue DNA and circulating cfDNA, though we did not use laser capture microdissection. Our *TSC2* mutation detection rate for specimens from patients with a pathological diagnosis was 71.4% (15/21) [see Additional file 3], coinciding with the average detection rate in previous NGS-based studies (70.7%, 53/75) [10–12, 15]. Admittedly, the absence of the laser capture microdissection method in this study may have resulted in potential mutations being overlooked. Regardless, the similar *TSC2* mutation detection rate supports the direct isolation of DNA from tissue as an acceptable approach for NGS-based genomic sequencing for S-LAM.

Regarding the *TSC2* mutation profile, no identical mutation was found among the patients, suggesting the high heterogenicity of *TSC2* gene alterations in S-LAM (Fig. 2), and more than half (13/20) of the detected mutations in *TSC2* were truncating mutations. Some studies have reported evidence of *TSC1* gene alterations in S-LAM [12, 14, 15]. Our study did not detect any mutation in *TSC1*, which supports the conventional notion that S-LAM is related to *TSC2*.

### TBLB samples are promising material for LAM genomic analysis

To our knowledge, previous LAM genomic studies have not used TBLB samples as a source of DNA. Nevertheless, our findings support this tissue as appropriate material for LAM genomic research for the following reasons. This study collected and tested 10 TBLB samples, 7 of which were found to carry *TSC2* mutations (70%), and surgically acquired samples did not yield a higher rate of *TSC2* mutation detection (69.2%, 9/13). Another intriguing finding is that *TSC2* mutations were confirmed in a TBLB-acquired specimen (lam6), even though this sample was not pathologically conclusive for LAM diagnosis. Current VEGF-D tests have spared most LAM patients from invasive biopsy procedures, and surgical sample availability has thus decreased. TBLB specimens are much more accessible and should be considered a promising source of tissue sample for LAM genomic testing, even when pathological findings are not highly indicative of LAM.

### Two samples from the same patient

Both lung (lam3-1) and renal AML (lam3-2) tissue from Patient LAM3 were tested, and an identical *TSC2* mutation was detected, supporting the “benign metastasis” theory [2]. However, the other mutation profiles of the two specimens differed strongly. This phenomenon may be explained by the evolution of LAM cells as the disease progresses. How LAM cells evolve remains unclear and understanding how cells change during the clinical course of LAM may provide solutions for overcoming rapamycin resistance.

### cfDNA is not an ideal material for NGS-based LAM genomic analysis

For many solid organ tumors, circulating cfDNA is commonly evaluated, yet data on cfDNA sequencing in LAM is limited. A previous study sequenced 61 cfDNA samples from S-LAM patients and found a point mutation in *TSC2* in one patient [26]; another investigation did not detect *TSC2* mutations in 22 LAM patients [14]. In the present study, only 1 in 22 patients was confirmed to carry a *TSC2* mutation. The low yield from cfDNA may be due to the low prevalence of LAM cells in the circulation. Hence, circulating cfDNA is not an ideal material for NGS-based sequencing in LAM.

### Mutation profiles and a novel *VEZF1* mutation

In addition to *TSC2*, mutations in other genes exhibit great variety. Notably, we identified a novel mutation common in 22 subjects. The mutation c.A659G in the *VEZF1* gene was detected in four patients: LAM1, LAM2, LAM11, and LAM14. The gene product vascular endothelial zinc finger 1 (Vezf1) is a Krüppel-like zinc finger protein that acts as a transcriptional activator of proangiogenic genes, blocking inappropriate promoter activation [27], and the *VEZF1* gene thus plays a vital role in vascular development. The mutation c.A659G is predicted to be “deleterious” and “high impact” by SIFT (Sorting Intolerant From Tolerant, https://sift.bii.a-star.edu.sg/) and Mutation Assessor (http://mutationassessor.org/r3/). One of the characteristic findings of LAM is a diseased lymphatic structure causing chylous effusions. The significance and role of *VEZF1* mutation are potential directions for future studies.

Clinical information for the four patents carrying *VEZF1* mutations is shown in Table 2. All four patients were first medically noticed due to pneumothorax. Overall, there is an established relationship of favorable prognosis and pneumothorax being the initial symptom compared to first presenting with exertional dyspnea [28]. Patients LAM1, -2, and -11 carried wildtype *TSC2* and presented with grade I or II lung lesions and well-preserved pulmonary function test (PFT) results, with FEV_1_%pred and DL_CO_ %pred being over 70%. Patient LAM14 was the only one of the four who also carried a *TSC2* mutation and had grade III lung lesions on HRCT, a distinctly shorter 6MWD and worse FEV_1_%pred and DL_CO_ %pred. We hypothesize that patients who carry *VEZF1* mutation but no detectable *TSC2* mutation may represent a more benign disease subtype, and *TSC2* mutation coexistence may indicate worse clinical outcomes.

### Patients with *TSC2* mutation have worse disease severity

As illustrated in Fig. 3, detectable *TSC2* mutation was related to significantly lower 6MWD, indicating more advanced functional deterioration. Patients harboring *TSC2* mutation were also more likely to have a VEGF-D value over 800 pg/mL (p < 0.05), and the average VEGF-D value was slightly higher (1821.9 pg/mL vs. 1498.4 pg/mL, p = 0.602) than in those without a detectable *TSC2* mutation. Though not statistically significant (*p* = 0.06), *TSC2* mutation was also assigned with a negative coefficient in the DL_CO_ predictive model in the multiple regression analysis. To sum up, a reasonable deduction is patients with detectable *TSC2* mutations would be more likely to present with worse disease severity.

### Stop-gain mutations in *TSC2* are related to a higher prevalence of pneumothorax

The function of mutation plays an important role in its pathogenicity. For example, a missense single-nucleotide variant alters only one amino acid, whereas a mutation that produces a premature stop codon would vastly change the length and hence the function of the protein product. In this study, five types of mutation functions in *TSC2* were detected, including missense, frameshift, nonframeshift deletion, splice site mutation, and stop-gain. Stop-gain mutations in *TSC2* were related to a higher rate of pneumothorax occurrence (Fig. 3). Genetic factors are considered to contribute to spontaneous pneumothoraces [29]. Our analysis further supports this correlation, and we found a more detailed relationship between a specific mutation function type and the prevalence of pneumothorax.

### Limitations

Our study has several limitations. Due to the limited number of patients recruited in the study, we were unable to produce a broader landscape of tumor-driver mutations in S-LAM. Interpretations of mutations were also limited to analysis based on public databases. Given the relatively small sample size, extra caution should be taken when interpreting the data, especially the correlations between mutation profile and clinical characteristics. More mechanistic studies are needed to better explain the influence and significance of genetic mutations. Additionally, patients with recent pneumothorax within less than three months were restricted from undergoing pulmonary function or functional tests, therefore we were limited when collecting a complete set of data of FEV_1_, DL_CO_, and 6MWD results in some patients. Finally, the clinical information included in this study was cross-sectional instead of longitudinal in nature, therefore we did not include more disease severity- and prognosis-related information, such as the rate of FEV_1_ decline.

## Conclusions

In this study, *TSC2* mutations were detected in 68.2% of 22 S-LAM patients, and TBLB samples were found to have potential as a tissue source for LAM genetic study. Additionally, an identical mutation, c.A659G, in *VEZF1* was found in four patients, which may be related to pneumothorax, representing a disease subtype of LAM with gentler disease severity. Patients with *TSC2* mutations tended to have worse clinical outcomes, with significantly lower 6MWD. Moreover, stop-gain mutation in *TSC2* was associated with a high rate of pneumothorax.

## Supplementary Information

**Additional file 1**. Target genes in the 301 tumor-driver genes panel used in this study.

**Additional file 2**. Clinical Information of the Subjects.

**Additional file 3**. Specimen Information and Sequencing Methods.

**Additional file 4**. Mutation Content Detected in this Study.

**Additional file 5**. Statistical Analysis Conducted in this Study.

## Data Availability

The clinical information and mutation content analysed in this study are included in this published article and its supplementary information files. The datasets generated and analysed during the current study are available in the Genome Sequence Archive for Human repository, https://ngdc.cncb.ac.cn/gsa-human/browse/HRA002652.

## Abbreviations

6MWD: Six-minute walk distance
6MWT: Six-minute walk test
AML: Angiomyolipoma
ATS: American Thoracic Society
BALF: Bronchoalveolar lavage fluid
cfDNA: Cell-free DNA
DL_CO_: Diffusing capacity for carbon monoxide
ELISA: Enzyme-linked immunosorbent assay
FEV_1_: Forced expiratory volume in 1 s
FFPE: Formalin-fixed paraffin-embedded
LAM: Lymphangioleiomyomatosis
LOH: Loss of heterozygosity
NGS: Next-generation sequencing
PCR: Polymerase chain reaction
PUMCH: Peking Union Medical College Hospital
S-LAM: Sporadic lymphangioleiomyomatosis
TBLB: Transbronchial lung biopsy
TSC: Tuberous sclerosis complex
VATS: Video-assisted thoracoscopy
VEGF-D: Vascular endothelial growth factor D
