# Supplementary material for: Gene mutations in sporadic lymphangioleiomyomatosis and genotype–phenotype correlation analysis
Source: BMC Pulm Med. 2022 Sep 18;22:354. doi: 10.1186/s12890-022-02154-0 (PMC9482747; doi:10.1186/s12890-022-02154-0)
Supplement: Supplementary file 1 — Additional file 1. Target genes in the 301 tumor-driver genes panel used in this study. [file 12890_2022_2154_MOESM1_ESM.docx]

Gene Symbol

| ABL1 |
| --- |
| ACVR1B |
| ACVR2A |
| AJUBA |
| AKT1 |
| AKT2 |
| ALK |
| AMER1 |
| ANTXR1 |
| APC |
| AR |
| ARAF |
| ARHGAP35 |
| ARID1A |
| ARID1B |
| ARID2 |
| ARID5B |
| ASXL1 |
| ATM |
| ATR |
| ATRX |
| AURKA |
| AXIN1 |
| AXIN2 |
| AXL |
| B2M |
| B4GALT3 |
| BAP1 |
| BRAD1 |
| BCL1 |
| BCL2L11 |
| BCOR |
| BCORL1 |
| BCYRN1 |
| BLM |
| BRAF |
| BRCA1 |
| BRCA2 |
| BRIP1 |
| BTK |
| C11orf30 |
| CARD11 |
| CASP8 |
| CBFB |
| CBL |
| CCND1 |
| CCNE1 |
| CD79A |
| CD79B |
| CDC73 |
| CDH1 |
| CDK12 |
| CDK4 |
| CDK6 |
| CDKN1A |
| CDKN1B |
| CDKN2A |
| CDKN2B |
| CDKN2C |
| CEBPA |
| CHEK2 |
| CIC |
| CREBBP |
| CRIPAK |
| CRLF2 |
| CSF1R |
| CTCF |
| CTNNB1 |
| CYLD |
| DAXX |
| DDR2 |
| DEPDC5 |
| DERL3 |
| DNMT1 |
| DNMT3A |
| DOT1L |
| DPYD |
| E2F3 |
| DGFR |
| EGR3 |
| EIF4A2 |
| ELF3 |
| EP300 |
| EPHA3 |
| EPHB6 |
| EPPK1 |
| ERBB2 |
| ERBB4 |
| ERCC2 |
| ERG |
| EZH2 |
| F2 |
| F5 |
| FAM46C |
| FANCA |
| FANCC |
| FANCD2 |
| FANCE |
| FANCF |
| FANCG |
| FANCL |
| FBXW7 |
| FGF10 |
| FGF14 |
| FGF19 |
| FGF23 |
| FGF3 |
| FGF4 |
| FGF6 |
| FGFR1 |
| FGFR2 |
| FGFR3 |
| FIP1L1 |
| FLT1 |
| FLT3 |
| FLT4 |
| FOXA1 |
| FOXA2 |
| FOXL2 |
| FUBP1 |
| FZR1 |
| GATA1 |
| GATA2 |
| GATA3 |
| GID3 |
| GNA11 |
| GNA13 |
| GNAQ |
| GNAS |
| GSK3B |
| H3F3A |
| H3F3B |
| H3F3C |
| HGF |
| HIST1H1C |
| HIST1H2BD |
| HIST1H3A |
| HIST1H3B |
| HIST1H3C |
| HIST1H3D |
| HIST1H3E |
| HIST1H3F |
| HIST1H3G |
| HIST1H3H |
| HIST1H3I |
| HIST1H3J |
| HNF1A |
| HRAS |
| IDH1 |
| IDH2 |
| IKZF1 |
| IL7R |
| IRF3 |
| JAK1 |
| JAK2 |
| JAK3 |
| JUN |
| KAT6A |
| KCNH2 |
| KDM5A |
| KDM5C |
| KDM6A |
| KDR |
| KEAP |
| KIT |
| KLF4 |
| KLHL6 |
| KMT2B |
| KMT2C |
| KMT2D |
| KRAS |
| LIFR |
| LMO1 |
| LRRFIP2 |
| LRRK2 |
| MAGI1 |
| MALAT1 |
| MAP2K1 |
| MAP2K2 |
| MAP2K4 |
| MAP3K1 |
| MAPK8IP1 |
| MDM2 |
| MDM4 |
| MECOM |
| MED12 |
| MEF2B |
| MEF2BNB |
| MEN1 |
| MET |
| MIR5096 |
| MLH1 |
| MPL |
| MRPL36 |
| MSH2 |
| MSH6 |
| MTOR |
| MUTYH |
| MYC |
| MYCL |
| MYCN |
| MYD88 |
| NAV3 |
| NCOA3 |
| NCOR1 |
| NF1 |
| NF2 |
| NFE2L2 |
| NFEL3 |
| NFKBIA |
| NKX2-1 |
| NOTCH1 |
| NOTCH2 |
| NPM1 |
| NPRL2 |
| NRAS |
| NSD1 |
| NTRK2 |
| NUP93 |
| PALB2 |
| PAX5 |
| PBRM1 |
| PCBP1 |
| PDGFRA |
| PDK1 |
| PDPK1 |
| PHF6 |
| PIK3CA |
| PIK3CG |
| PIK3R1 |
| PIK3R2 |
| POLQ |
| PPP2R1A |
| PRDM1 |
| PRX |
| PTCH1 |
| PTEN |
| PTPN11 |
| RAD21 |
| RAD50 |
| RAD51 |
| RB1 |
| RET |
| RNF2 |
| RNF43 |
| ROS1 |
| RPL22 |
| RPL5 |
| RUNX1 |
| SETBP1 |
| SETD2 |
| SF3B1 |
| SIN3A |
| SKP2 |
| SLTM |
| SMAD2 |
| SMAD4 |
| SMARCA4 |
| SMARCB1 |
| SMC1A |
| SMC3 |
| SMO |
| SMOX |
| SOCS1 |
| SOX17 |
| SOX9 |
| SPEN |
| SPOP |
| SRSF2 |
| STAG2 |
| STAT4 |
| STK11 |
| SUFU |
| TAF1 |
| TBL1XR1 |
| TBX3 |
| TERT |
| TET2 |
| TGFBR2 |
| TLR4 |
| TNFAIP3 |
| TNFRSF14 |
| TP53 |
| TPMT |
| TRAF7 |
| TSC1 |
| TSC2 |
| TSHR |
| TSHZ2 |
| TSHZ3 |
| U2AF1 |
| UFT1A1 |
| USP9X |
| VEGFA |
| VEZF1 |
| VHL |
| WISP3 |
| WT1 |
| XPO1 |
| ZNF217 |
| ZNF703 |

**Additional file 1. Target genes in the 301 tumore-driver genes panel used in this study.This chart listed the gene symbols of all the tested genes in the study.**
